# Kikuchi-Fujimoto disease in children: two case reports and a review of the literature

**DOI:** 10.1186/s13052-018-0522-9

**Published:** 2018-07-18

**Authors:** Mara Lelii, Laura Senatore, Ilaria Amodeo, Raffaella Pinzani, Sara Torretta, Stefano Fiori, Paola Marchisio, Samantha Bosis

**Affiliations:** 1 Pediatric Highly Intensive Care Unit, Department of Pathophysiology and Transplantation, Università degli Studi di Milano, Fondazione IRCCS Ca’ Granda Ospedale Maggiore Policlinico, Milan, Italy; 2 Neonatal Intensive Care Unit, Università degli Studi di Milano, Fondazione IRCCS Ca’, Granda Ospedale Maggiore Policlinico, Milan, Italy; 3 Division of Pathology, Department of Pathophysiology and Transplantation, Università degli Studi di Milano, Fondazione IRCCS Ca’ Granda Ospedale Maggiore Policlinico, Milan, Italy; 4 Department of Clinical Sciences and Community Health, Università degli Studi di Milano, Fondazione IRCCS Ca’ Granda Ospedale Maggiore Policlinico, Milan, Italy

**Keywords:** Kikuchi-Fujimoto disease, Lymphadenitis, Recurrent lymphadenitis, Persistent fever, Relapsing Kikuchi-Fujimoto disease, Hemophagocytic lymphohistiocytosis, HLH

## Abstract

**Background:**

Kikuchi-Fujimoto disease is a rare, idiopathic and generally self-limiting cause of lymphadenitis of unknow etiology with a low recurrence rate. The typical clinical signs are cervical lymphadenopathy, fever, and symptoms of respiratory infection, and less frequently chills, night sweats, arthralgia, rash, and weight loss.

**Case presentation:**

Here we describe two case reports of Kikuchi Fujimoto disease presenting in Milan within the space of a few months. The first involved the recurrence of KFD in a young boy from Sri Lanka; the second was a rare case of severe KFD complicated by HLH.

**Conclusions:**

Pediatricians must consider KFD in the differential diagnosis of fever of unknown origin in children, even in western countries. Although rare, recurrence and severe complications are possible. Where symptoms suggest KFD, a systematic diagnostic approach is key. Since no guidelines on the management of KFD are available, further studies should be conducted to investigate the therapeutic options and long term outcome in children.

## Background

Kikuchi-Fujimoto disease (FKD), also called histiocytic necrotizing lymphadenitis, is a rare, idiopathic and generally self-limiting cause of lymphadenitis. Prevalence of FKD is high among Japanese and other Asiatic individuals, but only isolated cases are reported in Europe. It was first described by the pathologists Kikuchi and Fujimoto in 1972 [1], but its etiology remains unclear, although the most likely theory suggests it is caused by one or more unidentified agents, probably viruses, that trigger a self-limiting autoimmune process in subjects with a genetic predisposition to autoimmune response [2, 3]. KFD is characterized by localized cervical lymphadenopathy, fever, and symptoms of upper respiratory tract infection, and less frequently chills, night sweats, arthralgia, rash, and weight loss. Lymph nodes are usually painful and tender; splenomegaly or hepatomegaly have sometimes been described. Although rare, KFD may present with neurologic involvement (both central and peripheral) [4]. Laboratory findings include high inflammatory marker levels (C reactive protein [CRP] and erythrocyte sedimentation rate [ESR]) and mild cytopenia [5, 6].

This clinical scenario is non-specific and can be mistaken for viral infection (such as mononucleosis), bacterial adenitis (mainly tuberculosis or cat scratch disease), malignant lymphoma or systemic lupus erythematosus (SLE). Several authors have also reported an association between FKD and SLE [5]. The diagnosis of KFD is confirmed by the histological analysis of an affected lymph node.

KFD is considered a benign disease, usually resolving within a few months. However, recurrences and complications including hemophagocytic lymphohistiocytosis (HLH) have been described in some patients. Here we report two cases of KFD presenting in Milan within the space of a few months. The first involved the recurrence of KFD in a young boy from Sri Lanka; the second was a rare case of severe KFD complicated by HLH.

## Case presentation 1

*Timeline*

**Table:** 

2012	In Sri Lanka diagnosis of KFD
January 2017	Admission to our hospital for fever of 4 days duration and bilateral cervical lymphadenopathy
Ten day after the admission	Lymph node biopsy was performed
Fifteen days after the admission	Stop of the fever
Twenty-two days after the admission	Discharged
Ten days after discharged	Follow up visit

A 12-year-old boy was admitted to our hospital with fever (38–39 °C) of 4 days’ duration and bilateral cervical lymphadenopathy. Five years earlier, while living in Sri Lanka, he had been admitted to the local hospital for intermittent fever of 12 days’ duration, mild cough, abdominal pain and significant bilateral cervical lymphadenopathy. Blood tests revealed very high LDH levels (2360 IU/L) and cytomegalovirus (CMV) antibodies (IgG and IgM). An abdomen ultrasound scan (US) was normal. Given the persistent fever and LDH levels, excisional biopsy of a cervical lymph node was performed, on the suspicion of a malignant lymphadenopathy. The histological analysis showed a lymphoid follicular hyperplasia with paracortical expansion and large areas containing immunoblasts, histiocytes and apoptotic cells, while atypical cells were absent. The clinical signs associated with the histological features suggested the diagnosis of KFD.

On his first examination after admission to our Emergency Pediatric Department, the patient was febrile and had a painful lymphadenopathy (3 cm in diameter) in the right side of the neck. His physical development was normal. Initial investigations revealed a mild increase in CRP (1.79 mg/dL) and hepatic enzymes (AST 51 IU, ALT 81 IU). Viral markers showed a past CMV infection while markers for Epstein Barr Virus (EBV), Toxoplasma gondii, Adenovirus and Parvovirus were negative. Angiotensin-converting enzyme (ACE) levels were normal. A chest X-ray was normal, while a neck US showed the presence of two hypoechoic and slightly inhomogeneous lymph nodes, with rich vascularized hilum and no evidence of colliquative phenomena. Some other small lymph nodes were also found in the submandibular region. Given the association of fever, cervical lymphadenopathies and mildly elevated inflammatory index, intravenous antibiotic therapy with cefotaxime (100 mg/kg/day) and analgesic oral therapy with paracetamol were started.

A tuberculin intradermal reaction was negative. As the blood tests confirmed the increase in hepatic enzymes, an abdomen US was performed, showing severe steatosis. Hepatitis B and C and HIV viral markers were negative; hepatic autoantibodies and LKM were negative, ANA and ASMA were mildly positive (ANA 1:80 homogeneous pattern, ASMA 1:80 vascular pattern), anti ds-DNA and the complement fraction (C3 and C4) were normal. Thyroid function, cholesterol (LDL and HDL) and A and B apolipoprotein were normal. Blood and urine copper and serum ceruloplasmin levels were normal. Urine galactose and fructose were normal.

**Fig. 1:**
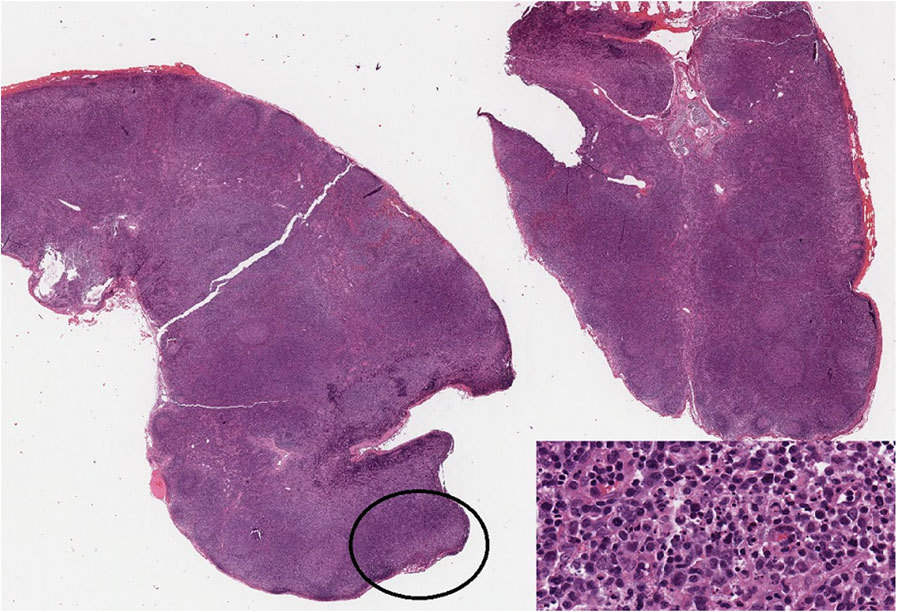
Case 1, 3rd level laterocervical lesion, including a lymphnode (1 cm in diameter) and several small tissue fragments (0.3 cm the largest) Lymph node (hematoxilin-eosin, 2×). The circle indicates the only small subcapsular necrotic focus. Inset shows a 20× magnification of the focus: note karyorrhectic debris and several large immunoblastic cells

**Fig. 2:**
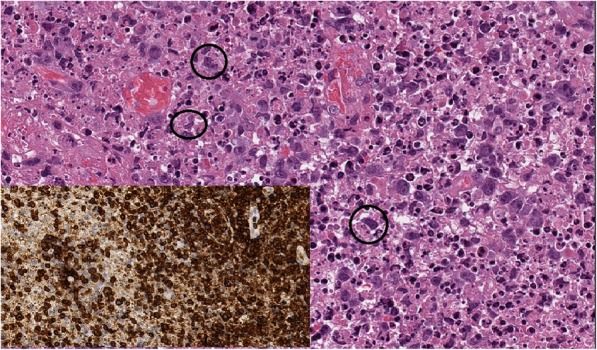
Detail of Case 1, tissue fragment (hematoxilin-eosin, 20×). Plasmacytoid histiocytes (arrows) and large immunoblastic cells in a necrotic and karyorrhectic background, devoid of granulocytes. Inset shows a CD3 immunohistochemical stain (20×), demonstrating that most of the large cells have a T-cell phenotype

Due to the persistence of intermittent fever, lymphadenopathy and the appearance of a diffuse erythematosus and itchy rash on the trunk, arms, and legs, an excisional lymph node biopsy was performed. The cytometric investigation showed B lymphocytes with non-malignant features, with just a reduced CD4 to CD8 lymphocyte ratio. The histologic exam revealed histiocytic necrotizing lymphadenitis, confirming the diagnosis of recurrent KFD (Fig. 1, Fig. 2). The microscopic and cultural exams were negative.

The intermittent fever lasted 15 days, then the patient’s general condition improved, with progressive complete normalization of blood tests. The boy was discharged without therapy. Seven days later, at the follow-up visit, he was still afebrile and in good general condition.

## Case presentation 2

*Timeline*

**Table:** 

April 2017	Admission to our hospital and start of cefotaxime therapy
Day after admission	Resolution of the fever
Six days after admission	Reappearance of fever, enlargement of the lymphadenopathies and worsening of the blood exams
Eleven days after admission	Cefotaxime was replaced by piperacillin/tazobactam (150 mg/kg/day) and vancomycin (40 mg/kg/day) with regression of fever
Twenty days after admission	The fever returned and progressive enlargement of the previous lymphadenopathies. An excisional cervical lymph node was performed.
Twenty-eight after admission	Diagnosis of initial HLH
A month after the admission	Diagnosis of posterior reversible encephalopathy syndrome (PRES)
A month and five days after the admission	Discharged
Ten days after discharged	Follow-up visit

A previously healthy 16-year-old Chinese girl came to the Emergency Pediatric Department with progressive fever, cough, headache and tender right cervical lymphadenopathy of four days’ duration. She also complained of intense fatigue and dizziness and had had two episodes of syncope. She was initially treated at home with oral amoxicillin/clavulanate for four days, without improvement. On physical exam, she had a tender right retro-angulo-mandibular lymphadenopathy of 3 cm in diameter, without hepatosplenomegaly, and hyperemic pharynx. An initial US revealed the presence of multiple bilateral lymphadenopathies of different sizes (the largest 2.7 cm) with inflammatory characteristics (hypo-anechoic and hypervascularized nodes with perilesional hyperechoic tissues). CRP was 2.06 mg/dL (normal value < 0.5 mg/dL). A complete blood count revealed leukopenia with a white blood cell count (WBC) of 3690 cells/mm^3^. A broad-spectrum intravenous antibiotic therapy was started (cefotaxime 100 mg/kg/day), with partial regression of the lymphadenopathies and resolution of the fever. After six days, she recommenced having two daily fevers of up to 41 °C with progressive enlargement of the lymphadenopathies, which became extremely painful. New tender bilateral lymphadenopathies appeared in the inguinal and axillary areas. Blood tests revealed a worsening leukopenia with WBC 1930 cells/mm^3^, neutrophils 760 cells/mm^3^, ESR 33 mm/h (normal value < 20 mm/h), ferritin 1303 ng/dL, LDH 857 U/L. Due to persistent dry cough, oral clarithromycin was started (15 mg/kg/day). A thorax X ray and abdominal US were normal, while lymph node US was unchanged. EBV, human immunodeficiency virus (HIV), CMV and multiple other viral, bacterial and fungal serum tests were all negative. Mantoux and Quantiferon Test were negative. Autoimmune lymphoproliferative syndrome was ruled out, due to the absence of α/β double-negative T cells.

Given her history suspicious for hematological disorders, bone marrow aspiration was performed, which revealed a slightly hypocellular marrow, with focal evidence of hemophagocytosis. Flow cytometry was negative for lymphoproliferative disorders and cytogenetic testing was normal. Perforin gene mutations were absent. Due to the persistence of high fever and painful lymphadenopathies, cefotaxime was replaced by piperacillin/tazobactam (150 mg/kg/day) and vancomycin (40 mg/kg/day). After 24 h, there was regression of the fever by lysis and regression of the systemic lymphadenopathies, associated with an improvement in inflammatory markers, except for persistent neutropenia.

**Fig. 3:**
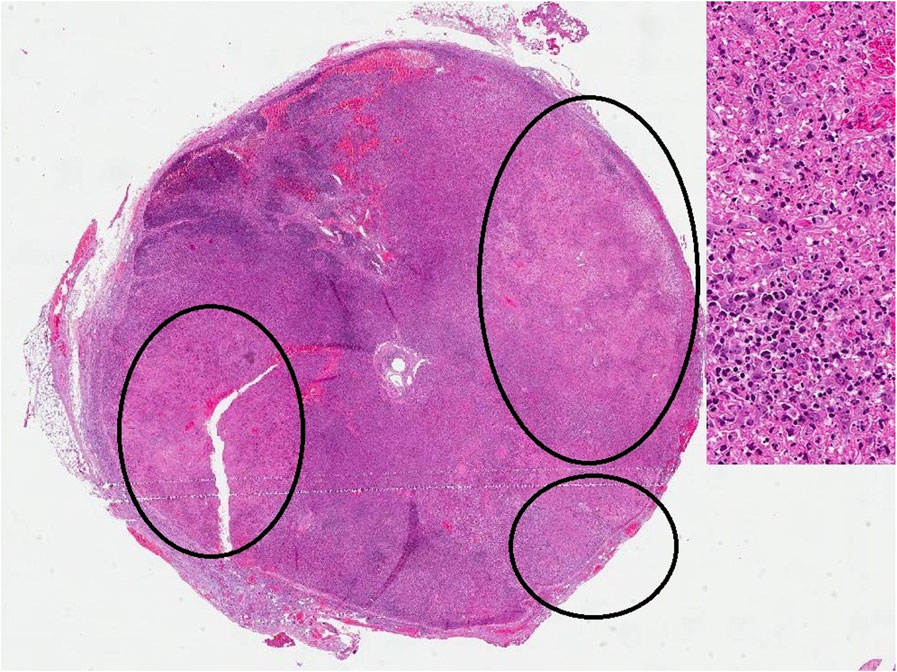
Case 2, a submandibular lymphnode (1,5 in diameter, hematoxilin-eosin, 2×). The circle indicates plurifocal subcapsular necrotic foci. Inset shows a 20× magnification of the largest focus: it was composed of fibrinoid material, karyorrhectic debris and several large immunoblastic cells

One week later, the fever returned and the patient reported intense fatigue, malaise and progressive enlargement of the previous lymphadenopathies, which were extremely painful and swollen. An excisional cervical lymph node biopsy was then performed, which was diagnostic for KFD (Fig. 3): as in case 1, the biopsy revealed wedge-shaped subcapsular necrotic foci composed of fibrinoid material with karyorrhectic debris, histiocytes with plasmacytoid features and crescentic nuclei, and several mitotically active immunoblasts; no granulocytic component was found. While in case 1 the finding was focal in the lymph node (that showed an overall preserved architecture with hyperplastic germinal centers) but extensive in the tissue fragments, in this case the lymph node was effaced by numerous necrotic foci. Immunohistochemistry revealed in both cases a T-cell phenotype (CD3+, CD2+, CD5+, CD7+, CD43+, CD8+ > CD4+) for the immunoblasts, along with focal and weak positivity for CD30, and a high proliferation index (Ki-67 / MIB-1 = 60%); ALK-1, EMA, CD25, CD56 and TdT were all negative. The B-cell markers CD20 and PAX5 highlighted only a few, scattered B-cells. Plasmacytoid histiocytes stained positively for CD68PG-M1 and CD123. In situ hybridization for Epstein-Barr virus was negative.

This histological picture suggested a T-cell neoplasm as the main differential diagnosis [6, 7]: however, the absence of T-cell antigen loss and of polyclonal T-cell receptor gamma chain rearrangement [8] were not indicative of a T-cell malignancy. Additionally, the clinical history, and the somewhat peculiar histological features (presence of a histiocytic component with typical plasmacytoid morphology in the background of fibrinoid necrosis, and absence of a granulocytic component), were indicative of KFD (relapsed in case 1) in the “proliferative” phase [9–12].

After 4 days, the fever spontaneously disappeared. However, inflammatory markers were still elevated, with alteration of liver enzymes and coagulation pattern (ALT 875 U/L, AST 342 U/L, LDH 1682 U/L, CRP 3.65 mg/dL, ferritin 9329 ng/mL, APTT 1.30, D-Dimer 2888 ng/mL, WBC 1500 cells/mm^3^, neutrophils 760 cells/mm^3^), suggestive of initial HLH. The patient was started on pulsed methylprednisolone therapy (1 g/day for 3 days) followed by prednisone (1.5 mg/kg/day) in combination with cyclosporine (4 mg/kg/day). A rapid improvement in her lymphadenopathies, fever and constitutional symptoms was seen soon after immunosuppressive therapy was started. A progressive normalization of blood analysis was also observed, with improvement of the leukopenia.

Although her symptoms improved with high doses of prednisone, the clinical course was complicated by the development of glucocorticoid-induced diabetes and hypertension. After four days of steroid treatment, the patient had a sudden rise in blood pressure (162/108 mmHg) associated with headache and malaise. Some hours later, she presented two generalized tonic-clonic seizures with cyanosis, requiring the administration of oxygen and intravenous diazepam. We performed an urgent computerized tomography (CT) brain scan, which was normal. Given the combination of generalized seizures, high blood pressure and immunosuppressive therapy, magnetic resonance imaging (MRI) of the brain was then performed, which showed pathological alterations suggestive of posterior reversible encephalopathy syndrome (PRES). An electroencephalogram (EEG) proved abnormal and antiepileptic therapy with Levetiracetam (750 mg/day) was started, achieving good control of the crisis. The hypertension was treated with amlodipine (10 mg/day). Follow-up brain MRI and EEG after 10 days showed a complete normalization of the previously described neurological alterations, thus confirming the diagnosis of PRES. Levetiracetam was then stopped and the anti-hypertensive therapy was gradually reduced. The patient continued high dose corticosteroids with a slow taper over several months in combination with cyclosporine and was discharged in good clinical condition.

## Discussion

Kikuchi-Fujimoto disease is a rare benign disease associated with histiocytic necrotizing lymphadenitis. It is endemic in Asia, especially among young women, but only isolated cases are reported in Europe and therefore its diagnosis may be difficult. FKD is characterized by lymphadenopathy with tenderness, night sweats and fever, less common manifestations include generalized lymphadenopathy, weight loss, splenomegaly, cutaneous rash, arthralgia, weight loss, nausea, vomiting and neurologic involvement. Although the lymphadenopathy usually affects the cervical lymph nodes, the axillary lymph nodes and other regions can also be affected. The affected lymph nodes are solid, movable, and painful but not suppurative; while the main affected node is usually solitary, multiple lymph nodes on both sides have been described [5, 6]. KFD is typically reported to have a self-limiting course, resolving within several months and with a recurrence rate of just 3 to 4% [13]. In our first patient, a young boy of Asian origin, KFD recurred five years after its first manifestation.

KFD remains an enigmatic disease, not only for its rarity and non-specific manifestations, but also because its etiology and pathogenesis remain unclear and there are no clear diagnostic criteria. Even though it is a benign condition, correct diagnosis is fundamental to rule out other causes of lymphadenopathy. Cervical lymphadenopathy is in fact common in the pediatric population, with about 38 to 45% of otherwise healthy children having palpable lymphadenopathy. For this reason, KFD can be mistaken for other common or more serious conditions, such as lymphoma or systemic lupus erythematosus - another hard-to-diagnose disease in children due to its widely varying symptoms [14].

The non-specific presentation requires numerous investigations. Excisional lymph node biopsy is the only reliable means of establishing a diagnosis, but a systematic approach is fundamental to exclude other causes of persistent fever and lymphadenitis and identify when biopsy is necessary. The starting diagnostic point is a collection of a careful medical history and a thorough physical examination. It is important to consider all symptoms (such as fever, anorexia, weight loss, night sweats, fatigue), their onset and duration, any insect bites, any changes in mass size, other recent illnesses or travel, exposure to animals, treatments (such as antibiotics) and response to them, vaccinations, family history, and the patient’s origins. Associated systemic symptoms, such as weight loss, night sweats, unexplained fever, or fatigue, suggest the need for further workup for possible malignancy or chronic inflammatory conditions. A careful examination should then be conducted to identify the number of masses, their location, size, mobility, tenderness, and characteristics of the masses on palpation and of the overlying skin. A complete physical examination of all body systems is necessary: infection is the most common cause of pediatric cervical lymphadenopathy and a benign reactive lymphadenopathy with infectious origin may be suggested by an associated illness (viral or bacterial) such as an upper respiratory infection, pharyngitis, tonsillitis, or otitis media [15].

While these data could orient towards a diagnosis, they are not unequivocal. The signs and symptoms of KFD are often enigmatic, especially in the early stages, and only their persistence can lead to diagnosis. For these reasons, some investigations need to be performed. Initial tests comprise complete blood cell count with WBC differential, erythrocyte sedimentation rate, liver enzymes and lactate dehydrogenase (significantly associated with malignancy), and inflammatory markers. Blood markers for virus infections that could cause lymphadenitis (CMV, HBV, *Herpes virus*, HIV, *Adenovirus*) and bacterial infections (*Toxoplasma gondii, Bartonella henselae, Borrelia burgorferi*) are also important [16]. Tuberculin skin test and Interferon-gamma release assay (IGRA), a pharyngeal swab and nasopharyngeal aspirate should also be performed.

A chest X-ray and neck US should be performed as a first step. Abdomen US could be useful to exclude the involvement of abdominal lymph nodes. In our patients, neck US features indicated benign or reactive lymph nodes. Fatty liver was found in case 1; this has never before been described in the manifestation of KFD. A cohort study should be conducted to evaluate hepatic US of these patients, although this finding was probably independent of the underlying disease.

If the initial investigation is inconclusive, or if indicated, additional investigations could include testing for *Listeria monocytogenes* and *Francisella tularensis* and ACE assay to exclude sarcoidosis. If, such as in our patients, symptoms persist and no definitive diagnosis has been arrived at, excisional lymph node biopsy should be performed [17].

In our first patient, biopsy was performed at an early stage because his history (a first occurrence of KFD five years before) and clinical course were very similar to the previous episode. However, other possible causes of lymphadenitis must be ruled out: since a low KFD recurrence rate is described in the literature, more frequent causes of lymphadenopathy should also be considered.

*Yoo IH* et al. found that a past history of other systemic illnesses and a higher Absolute Lymphocyte Count (ALC) despite a reduced WBC count were significantly associated with recurrent KFD, but there are insufficient data to predict recurrence [18]. Some data suggest that removal of the affected lymph node is not only diagnostic but also potentially therapeutic: after excisional lymph node biopsy some patients improve quickly [19], as did case 1 reported herein.

KFD has a wide clinical spectrum and is generally self-limiting. However, some patients suffer from prolonged systemic symptoms during the course of the disease, sometimes with severe complications. These include, especially in western countries, a few cases of secondary HLH, also known as hemophagocytic syndrome (HS) [13, 19–23]. HS is a histiocytic reactive process found in cases of strong immunologic activation such as severe infection, malignancy, autoimmune diseases, and metabolic diseases. It is characterized by histiocytic proliferation, hemophagocytosis, fever, hepatosplenomegaly, generalized lymphadenopathy, hypertriglyceridemia, and hypofibrinogenemia. KFD seems to be a hyperimmune reaction of immune cells to unidentified agents; too much stress could then lead to HLH [24–26]. The prognosis and therapeutic strategy for KFD in association with HS remains unclear. As *Kim* et al. conclude, childhood KFD is more frequently associated with HS and seems to have a less aggressive course and better prognosis than in adults [27].

No clear therapeutic guidelines are available on how to shorten the clinical course of KFD. Little information is available about the use of hydroxychloroquine or glucocorticoids [28, 29]. While we decided not to treat our first patient, who returned to good health after three weeks of fever, our second patient required prompt treatment because KFD associated with HLH has a potentially fatal outcome [27, 30]. It should be borne in mind that during the prolonged course of KFD, children suffer from impaired school performance, numerous medical visits and hospital admissions, and/or acute deterioration of health: further studies should be conducted to clarify possible therapeutic choices. Unfortunately, no clinical features that significantly affect the duration of KFD have been clearly identified, although some authors hypothesized that detection of atypical lymphocytes in peripheral blood may be a predictor for its duration [31, 32].

## Conclusion

Pediatricians must consider KFD in the differential diagnosis of fever of unknown origin in children, even in western countries: prelevalence of KFD is high among Asiatic individuals but until now only very rare cases are reported in Europe, however the immigration from Asian countries is likely to cause an increase of KFD cases. Although rare, recurrence and severe complications are possible. Where symptoms suggest KFD, a systematic diagnostic approach is key. Since no guidelines on the management of KFD are available, further studies should be conducted to investigate the therapeutic options and long term outcome in children.

## Abbreviations

ACE: Angiotensin-converting enzyme
ALC: Absolute lymphocyte count
CMV: Cytomegalovirus
CRP: C reactive protein
CT: Computerized tomography
EBV: Epstein Barr virus
EEG: Electroencephalogram
ESR: Erythrocyte sedimentation rate
HIV: Human immunodeficiency virus
HLH: Hemophagocytic lymphohistiocytosis
HS: Hemophagocytic syndrome
IGRA: Interferon-gamma release assay
KFD: Kikuchi-Fujimoto disease
MRI: Magnetic resonance imaging
PRES: Posterior reversible encephalopathy syndrome
SLE: Systemic lupus erythematosus
US: Ultrasound scan
WBC: White blood cell count
